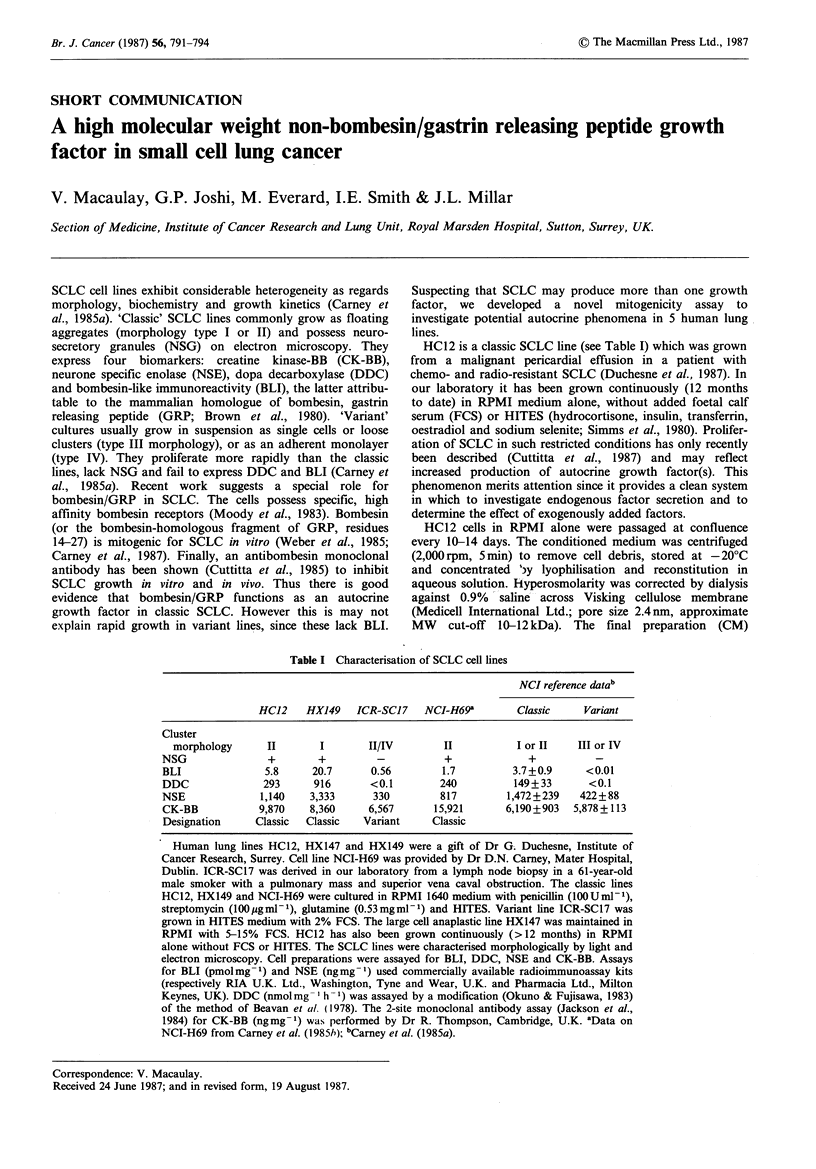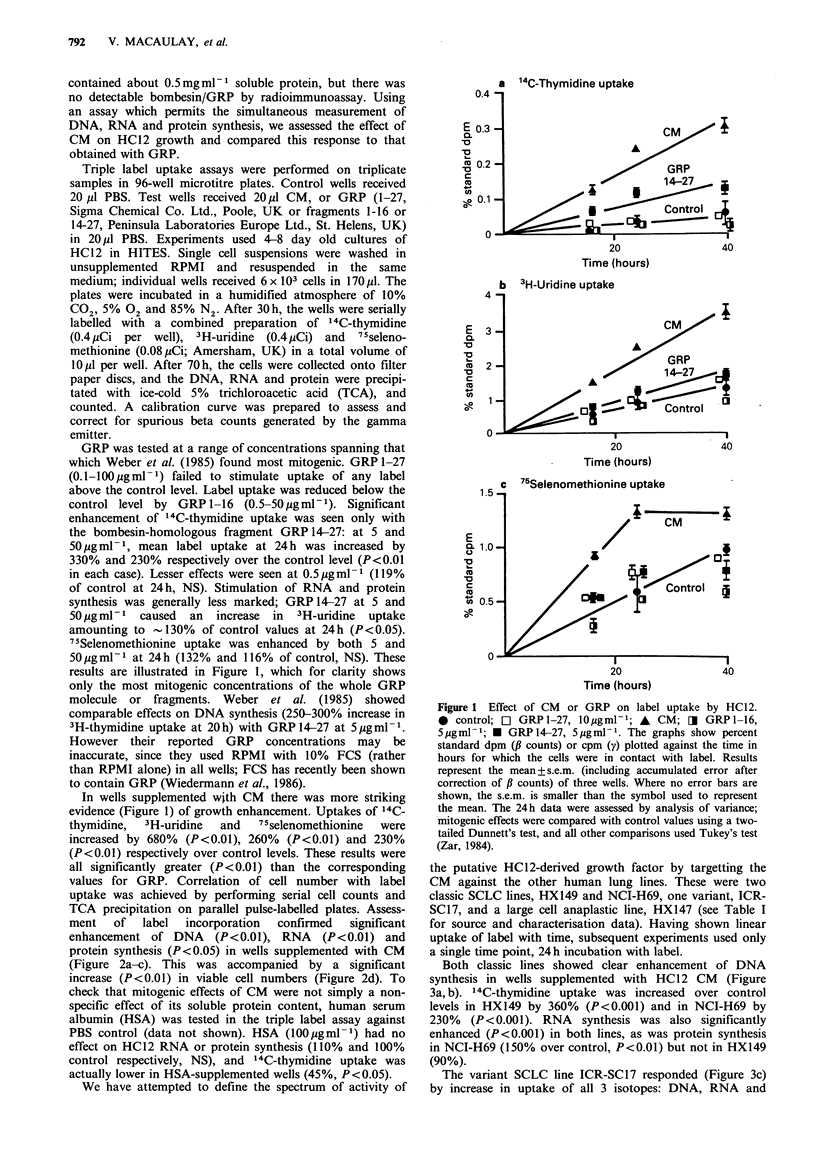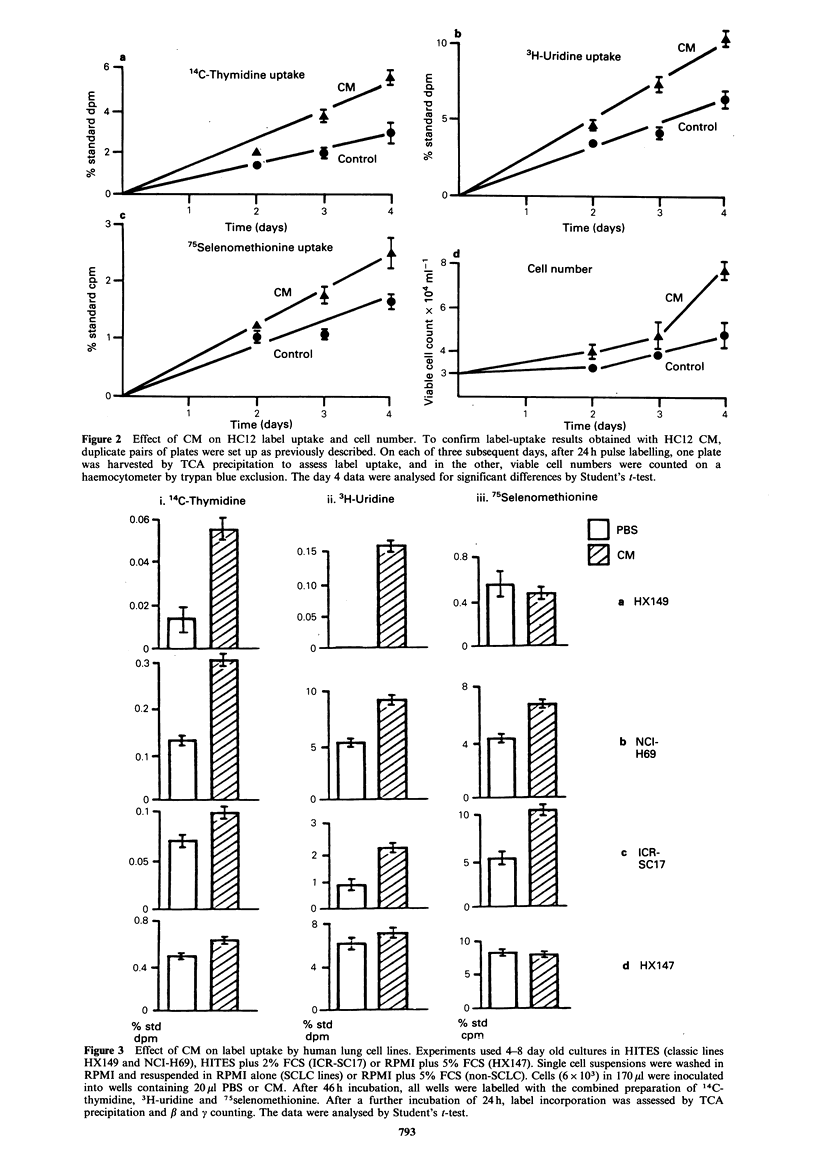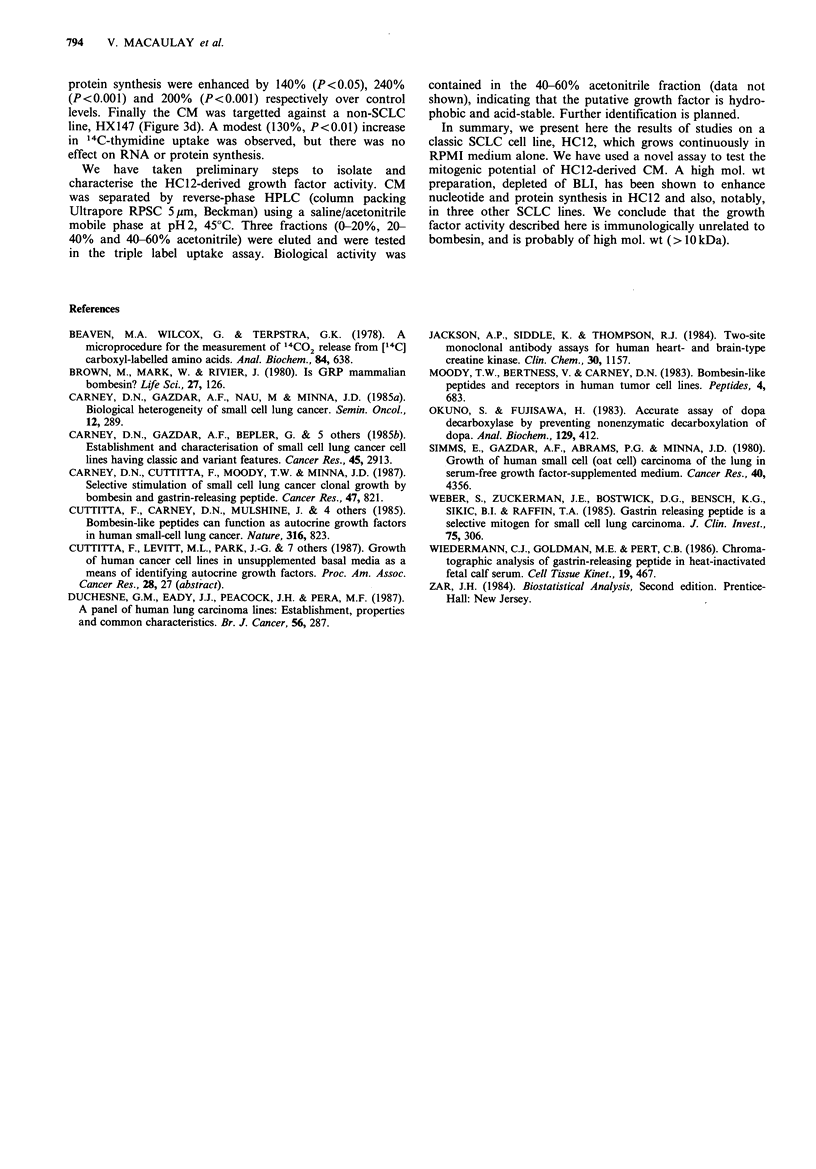# A high molecular weight non-bombesin/gastrin releasing peptide growth factor in small cell lung cancer.

**DOI:** 10.1038/bjc.1987.290

**Published:** 1987-12

**Authors:** V. Macaulay, G. P. Joshi, M. Everard, I. E. Smith, J. L. Millar

**Affiliations:** Institute of Cancer Research, Royal Marsden Hospital, Sutton, Surrey, UK.


Br. J. Cancer (1987) 56, 791–794 © The Macmillan Press Ltd., 1987

SHORT COMMUNICATION

### A high molecular weight non-bombesin/gastrin releasing peptide growth factor in small cell lung cancer

V. Macaulay, G.P. Joshi, M. Everard, I.E. Smith & J.L. Millar

*Section of Medicine, Institute of Cancer Research and Lung Unit, Royal Marsden Hospital, Sutton, Surrey, UK.*

SCLC cell lines exhibit considerable heterogeneity as regards morphology, biochemistry and growth kinetics (Carney *et al.*, 1985*a*). 'Classic' SCLC lines commonly grow as floating aggregates (morphology type I or II) and possess neurosecretory granules (NSG) on electron microscopy. They express four biomarkers: creatine kinase-BB (CK-BB), neurone specific enolase (NSE), dopa decarboxylase (DDC) and bombesin-like immunoreactivity (BLI), the latter attributable to the mammalian homologue of bombesin, gastrin releasing peptide (GRP; Brown *et al.*, 1980). 'Variant' cultures usually grow in suspension as single cells or loose clusters (type III morphology), or as an adherent monolayer (type IV). They proliferate more rapidly than the classic lines, lack NSG and fail to express DDC and BLI (Carney *et al.*, 1985*a*). Recent work suggests a special role for bombesin/GRP in SCLC. The cells possess specific, high affinity bombesin receptors (Moody *et al.*, 1983). Bombesin (or the bombesin-homologous fragment of GRP, residues 14–27) is mitogenic for SCLC *in vitro* (Weber *et al.*, 1985; Carney *et al.*, 1987). Finally, an antibombesin monoclonal antibody has been shown (Cuttitta *et al.*, 1985) to inhibit SCLC growth *in vitro* and *in vivo*. Thus there is good evidence that bombesin/GRP functions as an autocrine growth factor in classic SCLC. However this is may not explain rapid growth in variant lines, since these lack BLI. Suspecting that SCLC may produce more than one growth factor, we developed a novel mitogenicity assay to investigate potential autocrine phenomena in 5 human lung lines.

HC12 is a classic SCLC line (see Table I) which was grown from a malignant pericardial effusion in a patient with chemo- and radio-resistant SCLC (Duchesne *et al.*, 1987). In our laboratory it has been grown continuously (12 months to date) in RPMI medium alone, without added foetal calf serum (FCS) or HITES (hydrocortisone, insulin, transferrin, oestradiol and sodium selenite; Simms *et al.*, 1980). Proliferation of SCLC in such restricted conditions has only recently been described (Cuttitta *et al.*, 1987) and may reflect increased production of autocrine growth factor(s). This phenomenon merits attention since it provides a clean system in which to investigate endogenous factor secretion and to determine the effect of exogenously added factors.

HC12 cells in RPMI alone were passaged at confluence every 10–14 days. The conditioned medium was centrifuged (2,000 rpm, 5 min) to remove cell debris, stored at −20°C and concentrated by lyophilisation and reconstitution in aqueous solution. Hyperosmolarity was corrected by dialysis against 0.9% saline across Visking cellulose membrane (Medicell International Ltd.; pore size 2.4 nm, approximate MW cut-off 10–12 kDa). The final preparation (CM)

**Table I** Characterisation of SCLC cell lines

| | *HC12* | *HX149* | *ICR-SC17* | *NCI-H69*[a] | *NCI reference data*[b] | |
|---|---|---|---|---|---|---|
| | | | | | *Classic* | *Variant* |
| Cluster morphology | II | I | II/IV | II | I or II | III or IV |
| NSG | + | + | − | + | + | − |
| BLI | 5.8 | 20.7 | 0.56 | 1.7 | 3.7±0.9 | <0.01 |
| DDC | 293 | 916 | <0.1 | 240 | 149±33 | <0.1 |
| NSE | 1,140 | 3,333 | 330 | 817 | 1,472±239 | 422±88 |
| CK-BB | 9,870 | 8,360 | 6,567 | 15,921 | 6,190±903 | 5,878±113 |
| Designation | Classic | Classic | Variant | Classic | | |

Human lung lines HC12, HX147 and HX149 were a gift of Dr G. Duchesne, Institute of Cancer Research, Surrey. Cell line NCI-H69 was provided by Dr D.N. Carney, Mater Hospital, Dublin. ICR-SC17 was derived in our laboratory from a lymph node biopsy in a 61-year-old male smoker with a pulmonary mass and superior vena caval obstruction. The classic lines HC12, HX149 and NCI-H69 were cultured in RPMI 1640 medium with penicillin ($100\,U\,ml^{-1}$), streptomycin ($100\,\mu g\,ml^{-1}$), glutamine ($0.53\,mg\,ml^{-1}$) and HITES. Variant line ICR-SC17 was grown in HITES medium with 2% FCS. The large cell anaplastic line HX147 was maintained in RPMI with 5–15% FCS. HC12 has also been grown continuously (>12 months) in RPMI alone without FCS or HITES. The SCLC lines were characterised morphologically by light and electron microscopy. Cell preparations were assayed for BLI, DDC, NSE and CK-BB. Assays for BLI ($pmol\,mg^{-1}$) and NSE ($ng\,mg^{-1}$) used commercially available radioimmunoassay kits (respectively RIA U.K. Ltd., Washington, Tyne and Wear, U.K. and Pharmacia Ltd., Milton Keynes, UK). DDC ($nmol\,mg^{-1}\,h^{-1}$) was assayed by a modification (Okuno & Fujisawa, 1983) of the method of Beavan *et al.* (1978). The 2-site monoclonal antibody assay (Jackson *et al.*, 1984) for CK-BB ($ng\,mg^{-1}$) was performed by Dr R. Thompson, Cambridge, U.K. [a]Data on NCI-H69 from Carney *et al.* (1985*b*); [b]Carney *et al.* (1985*a*).

Correspondence: V. Macaulay.
Received 24 June 1987; and in revised form, 19 August 1987.

contained about 0.5 mg ml$^{-1}$ soluble protein, but there was no detectable bombesin/GRP by radioimmunoassay. Using an assay which permits the simultaneous measurement of DNA, RNA and protein synthesis, we assessed the effect of CM on HC12 growth and compared this response to that obtained with GRP.

Triple label uptake assays were performed on triplicate samples in 96-well microtitre plates. Control wells received 20 $\mu$l PBS. Test wells received 20 $\mu$l CM, or GRP (1-27, Sigma Chemical Co. Ltd., Poole, UK or fragments 1-16 or 14-27, Peninsula Laboratories Europe Ltd., St. Helens, UK) in 20 $\mu$l PBS. Experiments used 4–8 day old cultures of HC12 in HITES. Single cell suspensions were washed in unsupplemented RPMI and resuspended in the same medium; individual wells received $6 \times 10^3$ cells in 170 $\mu$l. The plates were incubated in a humidified atmosphere of 10% $CO_2$, 5% $O_2$ and 85% $N_2$. After 30 h, the wells were serially labelled with a combined preparation of $^{14}$C-thymidine (0.4 $\mu$Ci per well), $^3$H-uridine (0.4 $\mu$Ci) and $^{75}$selenomethionine (0.08 $\mu$Ci; Amersham, UK) in a total volume of 10 $\mu$l per well. After 70 h, the cells were collected onto filter paper discs, and the DNA, RNA and protein were precipitated with ice-cold 5% trichloroacetic acid (TCA), and counted. A calibration curve was prepared to assess and correct for spurious beta counts generated by the gamma emitter.

GRP was tested at a range of concentrations spanning that which Weber *et al.* (1985) found most mitogenic. GRP 1-27 (0.1–100 $\mu$g ml$^{-1}$) failed to stimulate uptake of any label above the control level. Label uptake was reduced below the control level by GRP 1-16 (0.5–50 $\mu$g ml$^{-1}$). Significant enhancement of $^{14}$C-thymidine uptake was seen only with the bombesin-homologous fragment GRP 14–27: at 5 and 50 $\mu$g ml$^{-1}$, mean label uptake at 24 h was increased by 330% and 230% respectively over the control level ($P<0.01$ in each case). Lesser effects were seen at 0.5 $\mu$g ml$^{-1}$ (119% of control at 24 h, NS). Stimulation of RNA and protein synthesis was generally less marked; GRP 14–27 at 5 and 50 $\mu$g ml$^{-1}$ caused an increase in $^3$H-uridine uptake amounting to ~130% of control values at 24 h ($P<0.05$). $^{75}$Selenomethionine uptake was enhanced by both 5 and 50 $\mu$g ml$^{-1}$ at 24 h (132% and 116% of control, NS). These results are illustrated in Figure 1, which for clarity shows only the most mitogenic concentrations of the whole GRP molecule or fragments. Weber *et al.* (1985) showed comparable effects on DNA synthesis (250–300% increase in $^3$H-thymidine uptake at 20 h) with GRP 14–27 at 5 $\mu$g ml$^{-1}$. However their reported GRP concentrations may be inaccurate, since they used RPMI with 10% FCS (rather than RPMI alone) in all wells; FCS has recently been shown to contain GRP (Wiedermann *et al.*, 1986).

In wells supplemented with CM there was more striking evidence (Figure 1) of growth enhancement. Uptakes of $^{14}$C-thymidine, $^3$H-uridine and $^{75}$selenomethionine were increased by 680% ($P<0.01$), 260% ($P<0.01$) and 230% ($P<0.01$) respectively over control levels. These results were all significantly greater ($P<0.01$) than the corresponding values for GRP. Correlation of cell number with label uptake was achieved by performing serial cell counts and TCA precipitation on parallel pulse-labelled plates. Assessment of label incorporation confirmed significant enhancement of DNA ($P<0.01$), RNA ($P<0.01$) and protein synthesis ($P<0.05$) in wells supplemented with CM (Figure 2a–c). This was accompanied by a significant increase ($P<0.01$) in viable cell numbers (Figure 2d). To check that mitogenic effects of CM were not simply a non-specific effect of its soluble protein content, human serum albumin (HSA) was tested in the triple label assay against PBS control (data not shown). HSA (100 $\mu$g ml$^{-1}$) had no effect on HC12 RNA or protein synthesis (110% and 100% control respectively, NS), and $^{14}$C-thymidine uptake was actually lower in HSA-supplemented wells (45%, $P<0.05$).

We have attempted to define the spectrum of activity of

**Figure 1** Effect of CM or GRP on label uptake by HC12. ● control; □ GRP 1-27, 10 $\mu$g ml$^{-1}$; ▲ CM; ◧ GRP 1-16, 5 $\mu$g ml$^{-1}$; ■ GRP 14-27, 5 $\mu$g ml$^{-1}$. The graphs show percent standard dpm ($\beta$ counts) or cpm ($\gamma$) plotted against the time in hours for which the cells were in contact with label. Results represent the mean ± s.e.m. (including accumulated error after correction of $\beta$ counts) of three wells. Where no error bars are shown, the s.e.m. is smaller than the symbol used to represent the mean. The 24 h data were assessed by analysis of variance; mitogenic effects were compared with control values using a two-tailed Dunnett's test, and all other comparisons used Tukey's test (Zar, 1984).

the putative HC12-derived growth factor by targetting the CM against the other human lung lines. These were two classic SCLC lines, HX149 and NCI-H69, one variant, ICR-SC17, and a large cell anaplastic line, HX147 (see Table I for source and characterisation data). Having shown linear uptake of label with time, subsequent experiments used only a single time point, 24 h incubation with label.

Both classic lines showed clear enhancement of DNA synthesis in wells supplemented with HC12 CM (Figure 3a, b). $^{14}$C-thymidine uptake was increased over control levels in HX149 by 360% ($P<0.001$) and in NCI-H69 by 230% ($P<0.001$). RNA synthesis was also significantly enhanced ($P<0.001$) in both lines, as was protein synthesis in NCI-H69 (150% over control, $P<0.01$) but not in HX149 (90%).

The variant SCLC line ICR-SC17 responded (Figure 3c) by increase in uptake of all 3 isotopes: DNA, RNA and

**Figure 2** Effect of CM on HC12 label uptake and cell number. To confirm label-uptake results obtained with HC12 CM, duplicate pairs of plates were set up as previously described. On each of three subsequent days, after 24 h pulse labelling, one plate was harvested by TCA precipitation to assess label uptake, and in the other, viable cell numbers were counted on a haemocytometer by trypan blue exclusion. The day 4 data were analysed for significant differences by Student's *t*-test.

**Figure 3** Effect of CM on label uptake by human lung cell lines. Experiments used 4–8 day old cultures in HITES (classic lines HX149 and NCI-H69), HITES plus 2% FCS (ICR-SC17) or RPMI plus 5% FCS (HX147). Single cell suspensions were washed in RPMI and resuspended in RPMI alone (SCLC lines) or RPMI plus 5% FCS (non-SCLC). Cells ($6 \times 10^3$) in 170 $\mu$l were inoculated into wells containing 20 $\mu$l PBS or CM. After 46 h incubation, all wells were labelled with the combined preparation of $^{14}$C-thymidine, $^3$H-uridine and $^{75}$selenomethionine. After a further incubation of 24 h, label incorporation was assessed by TCA precipitation and $\beta$ and $\gamma$ counting. The data were analysed by Student's *t*-test.

protein synthesis were enhanced by 140% ($P<0.05$), 240% ($P<0.001$) and 200% ($P<0.001$) respectively over control levels. Finally the CM was targetted against a non-SCLC line, HX147 (Figure 3d). A modest (130%, $P<0.01$) increase in $^{14}$C-thymidine uptake was observed, but there was no effect on RNA or protein synthesis.

We have taken preliminary steps to isolate and characterise the HC12-derived growth factor activity. CM was separated by reverse-phase HPLC (column packing Ultrapore RPSC 5 $\mu$m, Beckman) using a saline/acetonitrile mobile phase at pH 2, 45°C. Three fractions (0–20%, 20–40% and 40–60% acetonitrile) were eluted and were tested in the triple label uptake assay. Biological activity was contained in the 40–60% acetonitrile fraction (data not shown), indicating that the putative growth factor is hydrophobic and acid-stable. Further identification is planned.

In summary, we present here the results of studies on a classic SCLC cell line, HC12, which grows continuously in RPMI medium alone. We have used a novel assay to test the mitogenic potential of HC12-derived CM. A high mol. wt preparation, depleted of BLI, has been shown to enhance nucleotide and protein synthesis in HC12 and also, notably, in three other SCLC lines. We conclude that the growth factor activity described here is immunologically unrelated to bombesin, and is probably of high mol. wt (>10 kDa).

### References

BEAVEN, M.A. WILCOX, G. & TERPSTRA, G.K. (1978). A microprocedure for the measurement of $^{14}CO_2$ release from [$^{14}$C] carboxyl-labelled amino acids. *Anal. Biochem.*, **84**, 638.

BROWN, M., MARK, W. & RIVIER, J. (1980). Is GRP mammalian bombesin? *Life Sci.*, **27**, 126.

CARNEY, D.N., GAZDAR, A.F., NAU, M & MINNA, J.D. (1985*a*). Biological heterogeneity of small cell lung cancer. *Semin. Oncol.*, **12**, 289.

CARNEY, D.N., GAZDAR, A.F., BEPLER, G. & 5 others (1985*b*). Establishment and characterisation of small cell lung cancer cell lines having classic and variant features. *Cancer Res.*, **45**, 2913.

CARNEY, D.N., CUTTITTA, F., MOODY, T.W. & MINNA, J.D. (1987). Selective stimulation of small cell lung cancer clonal growth by bombesin and gastrin-releasing peptide. *Cancer Res.*, **47**, 821.

CUTTITTA, F., CARNEY, D.N., MULSHINE, J. & 4 others (1985). Bombesin-like peptides can function as autocrine growth factors in human small-cell lung cancer. *Nature*, **316**, 823.

CUTTITTA, F., LEVITT, M.L., PARK, J.-G. & 7 others (1987). Growth of human cancer cell lines in unsupplemented basal media as a means of identifying autocrine growth factors. *Proc. Am. Assoc. Cancer Res.*, **28**, 27 (*abstract*).

DUCHESNE, G.M., EADY, J.J., PEACOCK, J.H. & PERA, M.F. (1987). A panel of human lung carcinoma lines: Establishment, properties and common characteristics. *Br. J. Cancer*, **56**, 287.

JACKSON, A.P., SIDDLE, K. & THOMPSON, R.J. (1984). Two-site monoclonal antibody assays for human heart- and brain-type creatine kinase. *Clin. Chem.*, **30**, 1157.

MOODY, T.W., BERTNESS, V. & CARNEY, D.N. (1983). Bombesin-like peptides and receptors in human tumor cell lines. *Peptides*, **4**, 683.

OKUNO, S. & FUJISAWA, H. (1983). Accurate assay of dopa decarboxylase by preventing nonenzymatic decarboxylation of dopa. *Anal. Biochem.*, **129**, 412.

SIMMS, E., GAZDAR, A.F., ABRAMS, P.G. & MINNA, J.D. (1980). Growth of human small cell (oat cell) carcinoma of the lung in serum-free growth factor-supplemented medium. *Cancer Res.*, **40**, 4356.

WEBER, S., ZUCKERMAN, J.E., BOSTWICK, D.G., BENSCH, K.G., SIKIC, B.I. & RAFFIN, T.A. (1985). Gastrin releasing peptide is a selective mitogen for small cell lung carcinoma. *J. Clin. Invest.*, **75**, 306.

WIEDERMANN, C.J., GOLDMAN, M.E. & PERT, C.B. (1986). Chromatographic analysis of gastrin-releasing peptide in heat-inactivated fetal calf serum. *Cell Tissue Kinet.*, **19**, 467.

ZAR, J.H. (1984). *Biostatistical Analysis*, Second edition. Prentice-Hall: New Jersey.